# C1QBP regulates apoptosis of renal cell carcinoma via modulating xanthine dehydrogenase (XDH) mediated ROS generation

**DOI:** 10.7150/ijms.71703

**Published:** 2022-05-09

**Authors:** Yiting Wang, Shuang Liu, Shaoping Tian, Runxuan Du, Tianyu Lin, Xuesong Xiao, Rui Wang, Ruibing Chen, Hua Geng, Saravanan Subramanian, Yuanjie Niu, Yong Wang, Dan Yue

**Affiliations:** 1 Department of Microbiology, School of Medical Laboratory, Tianjin Medical University, Tianjin 300203, China; 2 The Second Hospital of Tianjin Medical University, Tianjin Institute of Urology, Tianjin Medical University, Tianjin 300211, China; 3 Department of Clinical Laboratory, Tianjin Children's Hospital/Tianjin University Children's Hospital, Tianjin 300134, China; 4 School of Pharmaceutical Science and Technology, Tianjin University, Tianjin 300072, China; 5 Center for Intestinal and Liver Inflammation Research, Stanley Manne Children's Research Institute, Ann & Robert H. Lurie Children's Hospital of Chicago, Chicago, Illinois, USA; 6 Department of Pediatrics, Feinberg School of Medicine, Northwestern University, Chicago, Illinois, USA

**Keywords:** Apoptosis, C1QBP, ROS, Renal cell carcinoma, XDH

## Abstract

**Background:** Complement component 1 Q subcomponent binding protein (C1QBP) plays a vital role in the progression and metabolism of cancer. Studies have shown that xanthine dehydrogenase (XDH)-derived reactive oxygen species (ROS) accelerates tumor growth, and also induces mutations or produces cytotoxic effects concurrently. However, the role of C1QBP in metabolism, oxidative stress, and apoptosis of renal cell carcinoma (RCC) cells have not yet been explored.

**Methods:** Metabolomics assay was applied to investigate the role of C1QBP in RCC metabolism. C1QBP knockdown and overexpression cells were established via lentiviral infection and subjected to apoptosis and ROS assay *in vitro*. RNA stability assay was applied to characterize the mechanism of *C1QBP* regulating *XDH* transcription. *In vivo*, orthotopic tumor xenografts assay was performed to investigate the role of C1QBP in RCC progression.

**Results:** Metabolomics investigation revealed that C1QBP dramatically diminished the hypoxanthine content in RCC cells. C1QBP promoted the mRNA and protein expression of hypoxanthine catabolic enzyme XDH. Meanwhile, *C1QBP* may affect *XDH* transcription by regulating the mRNA level of *XDH* transcriptional stimulators *IL-6*, *TNF-α*, and *IFN-γ*. Moreover, the expression of C1QBP and XDH was lower in RCC tumors compared with the tumor-associated normal tissues, and their down-regulation was associated with higher Fuhrman grade. C1QBP significantly increased ROS level, apoptosis, and the expression of apoptotic proteins such as cleaved caspase-3 and bax/bcl2 via regulating XDH.

**Conclusion:** C1QBP promotes the catabolism of hypoxanthine and elevates the apoptosis of RCC cells by modulating XDH-mediated ROS generation.

## Introduction

Renal cell carcinoma is one of the most common cancers in the urinary system, causing over 140,000 deaths worldwide each year^1^. Clear cell RCC (ccRCC) is the most common subtype among the malignant RCCs, accounting for 70-80%, followed by papillary (15%), chromophobe (4-5%), and other fewer subtypes^2^. The onset of RCC is hidden, and 16% of patients have metastasized at first diagnosis with a 5-year survival of 9-12%^3^. Traditional chemotherapy and radiotherapy have limited effect in RCC, and molecular biomarkers are more targetable to guide treatment decisions. Recently, with the advent of immunotherapy and anti-angiogenesis therapy, the survival of RCC patients has been improved^4^. Despite the progress of available therapeutic agents, significant challenges remain regarding patient selection and optimal therapeutic combinations.

C1QBP, also known as HABP1, SF2p32, gC1qR, and p32, is a highly conservative and multi-functional protein. It ubiquitously distributes in the mitochondria matrix, cell membrane, as well as in the secreted form, and plays a vital role in inflammation, infection, and cancer^5^. A few studies have shown that C1QBP is overexpressed in malignant cells, e.g. breast, colon, and gastric cancers, and promotes various cellular processes, including cell survival, growth, apoptosis, and metastasis^5^,^6^,^7^,^8^. Interestingly, in our previous study, unlike other malignancies, C1QBP showed diminished expression in RCC compared with the corresponding adjacent normal kidney tissues and suppressed adhesion and metastasis of RCC^9^. Besides, C1QBP is essential for metabolism balance, which maintains oxidative phosphorylation (OXPHOS) that produces ROS with the potential to damage cells^10^. Furthermore, C1QBP regulates metabolism reprogramming to maintain the balance between OXPHOS and glycolysis in breast cancer^11^. However, the underlying mechanism of C1QBP regulation in the metabolism and progression of RCC is ill-defined.

Xanthine dehydrogenase, also known as xanthine oxidoreductase (XOR) and xanthine oxidase (XO), is the key enzyme for purine catabolism. XDH catalyzed the oxidation of hypoxanthine to xanthine and further catalyzed the formation of uric acid and ROS from xanthine^12^. XDH can also oxidize different endogenous metabolites and various exogenous substances to produce ROS, toxic substance, and anticancer drugs^13^. Studies have shown that XDH-derived ROS causes endothelial dysfunction, promotes inflammation, accelerates tumor growth, and also induces mutation or produces cytotoxic effect concurrently^14^. ROS accumulation activates Fas and tumor necrosis factor receptor 1 (TNFR1) receptors to bind to their respective ligands, followed by triggering caspase promoter caspase-8 activation and downstream bax expression, mitochondrial cytochrome c release, and caspase-3 activation, leading to cell death^15^.

In the present study, we investigated the role of C1QBP in regulating purine metabolism and progression of RCC *in vitro* and *in vivo*. Our results showed that C1QBP regulated the catabolism of hypoxanthine and apoptosis in RCC cells by modulating XDH mediated ROS generation.

## Materials and Methods

### Cell culture and establishment of stable cell lines

Human embryonic kidney cell HEK-293 T and human RCC cell lines 786-O and ACHN were obtained from American Type Culture Collection (ATCC, USA). The cells were grown in Dulbecco's Modified Eagle's Medium (DMEM) and Eagle's Minimum Essential Medium (MEM) (HAKATA, China) supplemented with 10% fetal bovine serum (HAKATA, China) and 1 × penicillin/streptomycin (Biological Industries, Israel) in a humidified 37°C incubator in the presence of 5% CO_2_.

Our group had completed the construction and identification of C1QBP lentiviral interference vector in previous work, the three primer sequences of pLKO.1-shC1QBP were listed in Supplemental Table 1. We transfected successful pLKO.1-shC1QBP vector into renal cancer cells, and selected the one with the best knockdown efficiency using quantitative real-time PCR (qRT-PCR) and western blot technology. To construct C1QBP overexpression and knocked-down stable cell lines, HEK-293 T cells were co-transfected with lentiviral vectors (Promega, USA), including pLKO.1-Scr, pLKO.1-shC1QBP, pCDH or pCDH-C1QBP and with packaging plasmid system consisted of pSEV-REV, pMD 2.G and pRRE for 48 h using lipofectamine 2000 (Invitrogen, USA). The lentivirus supernatant was collected, centrifuged, and filtered with a 0.45 μm filter. Then 786-O and ACHN cells were infected with the lentivirus supernatant respectively for 48 h and selected for 10 days with 2 μg/ml puromycin (Sangon Biotech, China) or 100 mg/ml G418 (MDBio Inc, China), subsequently maintained with 0.5 μg/ml puromycin or 25 mg/ml G418. The expression of C1QBP in the stable cell lines was determined using qRT-PCR and western blot.

### RNA extraction, reverse transcription and quantitative real-time PCR

Total RNA extraction, reverse transcription, and qRT‐PCR analysis were performed as previously described^9^. The primers for qRT‐PCR analysis were synthesized by Hua Da Gene. The primer sequences were shown in Table 1. The expression of target gene was normalized to GAPDH and the data were analyzed by the 2^-ΔΔCt^ method.

### Western blot

Proteins were extracted with SDS lysis buffer and 1 × protease inhibitor cocktail (Roche, Germany), and the concentrations were measured by Enhanced BCA Protein assay kit (Thermo Fisher Scientific, USA). Proteins were separated by SDS-PAGE, then transferred onto a PVDF membrane (Millipore, USA), followed by blocking with 5% milk (BD, USA) in TBST (Tris‐buffered saline with Tween‐20) solution for 1.5 h at room temperature. The membrane was incubated at 4°C overnight with corresponding primary antibodies to C1QBP (Abcam, ab101267; 1:2000), XDH (Affinity Biosciences, DF8111; 1:1000), caspase-3 (Abcam, ab184787; 1:2000), bcl2 (Abcam, ab182858; 1:2000), bax (Abcam, ab32503; 1:2000) and β-actin (Affinity Biosciences, T0022; 1:3000). After washing three times in TBST, the membranes were further incubated with HRP‐conjugated goat anti‐rabbit IgG (Affinity Biosciences, S0001; 1:5000) or goat anti‐mouse IgG (Affinity Biosciences, S0002; 1:4000) secondary antibodies for 1 h at room temperature and developed by ECL Western Blotting Detection Reagents (Millipore, USA). The expression of target protein was normalized with β-actin.

### Metabolomics relative-quantitative Analysis

Well-growth and logarithmic 786-O C1QBP overexpression and control cells (1 × 10^7^) were washed twice with ice-cold PBS, and then washed cells with ice-cold saline (0.9% sodium chloride solution) quickly, discarding the supernatant completely after each wash. Finally, cells were collected in a 1.5 ml centrifuge tube with 1ml of methanol/acetonitrile/water (2:2:1, v/v/v) and stored at -80°C after quick freezing with liquid nitrogen.

The samples were thawed and mixed with 1 ml cold methanol/acetonitrile/water (2:2:1, v/v/v) and sonicated at low temperature (30 min/once, twice). Followed by centrifugation for 20 min (14000 g, 4°C), and the supernatant was dried in a vacuum centrifuge. For LC-MS analysis, the samples were re-dissolved in 100 μl acetonitrile/water (1:1, v/v) and adequately vortexed, and then centrifuged (14000 g, 4°C, 15 min). The supernatants were collected for LC-MS/MS analysis using a UHPLC (1290 Infinity LC, Agilent Technologies) coupled to a triple quadrupole mass spectrometer.

MRM Analyzer software was used to extract the chromatographic peak area and retention time. Detected metabolites in pooled samples with the coefficient of variation (CV) less than 30% were denoted as reproducible measurements. To find the differently expressed metabolites, statistical analyses between two sample groups were performed by calculating the fold changes and *P* values of metabolites using Student *t*-test, *P* < 0.05, fold change > 1.5, were marked as the significantly changed metabolites between sample groups.

### Xanthine/hypoxanthine detection

Xanthine/hypoxanthine level was detected by using Xanthine/Hypoxanthine Colorimetric/Fluorometric Assay Kit (Bio Vision, USA) in RCC cells. According to the manufacturer's protocol, cells (1 × 10^6^) were lysed with 100 μl ice-cold Xanthine Assay Buffer for 10 min on ice, centrifuged at 12000 rpm for 5 min, followed by collecting the supernatant. The mixture of samples and reaction buffer were incubated for 30 min at room temperature, protected from light. Measured absorbance at λ = 570 nm using a Microplate reader. Xanthine/hypoxanthine concentration (nmol/ml) was calculated in the samples according to the standard curve.

### Human patients

Human patient cohorts were used to explore the clinical importance of C1QBP and XDH expression in RCC. We collected 57 pairs of primary RCC tissues and corresponding para-carcinoma tissues. All RCC tissues were surgically removed and paraffin-embedded in the Second Hospital of Tianjin Medical University between January 2013 and December 2018 with the patients' consent and approval from the ethical committee. Criteria for inclusion were that patients with pathological diagnosis of RCC and patients had undergone radical nephrectomy with no preoperative and postoperative adjuvant therapy. In addition, patient clinical-pathological parameters were collected including gender, age, tumor size, TNM stage, and Fuhrman grade.

### IHC staining

Tissues were formalin-fixed and embedded in paraffin blocks and then cut into 5 µm thickness sections. Tissue sections on glass slides were deparaffinized in xylene, and dehydrated in gradient ethanol, followed by blocking of endogenous peroxidase. Repaired antigens with boiling citrate buffer and blocked non-specific background staining with 5% BSA (Solarbio, China). The tissue sections were incubated with primary antibody C1QBP (Santa Cruz Biotechnology, USA) or XDH (Abcam, USA) overnight at 4°C. On the second day, after washing with PBS, the slides were immunoblotted using a Universal kit (mouse/rabbit polymer detection system) (ZSGB.BIO, China). Sections were developed by peroxidase substrate DAB Detection Kit (ZSGB.BIO, China) and then counterstained by hematoxylin. Finally, slides were covered with neutral resin.

All immunostaining slides were scored by two independent researchers for the percentage and intensity of cells showing specific immunostaining signals. The score of percentage of positively stained cells was evaluated from 0 to 5: 0, (0-1)% cells positive; 1, (1-5)% cells positive; 2, (6-10)% cells positive; 3, (11-20)% cells positive; 4, (21-50)% cells positive; and 5, (51-100)% of cells positive. The score of immunostaining intensity as follows: 0, no staining; 1, yellow-brown staining; 2, brown staining. The final score was calculated by adding the percentage and intensity scores and recorded as negative (0-3) and positive (4-7).

### RNA stability

RCC cells were treated with 5 μg/ml actinomycin D (Med Chem Express, USA) at the indicated time point 0 min, 15 min, 30 min, 45 min, and 60 min. The RNA was extracted using Trizol (Ambion, USA) and detected *XDH* mRNA level by qRT-PCR.

### Reactive oxygen species (ROS) Assay

The cells were detected with ROS Assay Kit (Beyotime Biotechnology, China) according to the manufacturer's instruction. RCC cells were seeded at a density of 3 × 10^5^ cells/well in a 6-well plate and cultured overnight. The cells were treated with 10 μM DCFH-DA and incubated at 37°C for 20 min. Subsequently, washing cells three times to fully remove the DCFH-DA from liquid. In the positive group, cells were treated with 50 mg/ml Rosup for 20 min. Later, cells were collected, ROS were detected by a fluorescent microplate reader at λ = 488 nm/525 nm or detected via flow cytometry.

### Annexin V/PI apoptosis assay

Renal cancer cells were detected with an Annexin V-FITC apoptosis assay kit (Absin bioscience, China) according to the manufacturer's instruction. The cells were harvested and counted, and 5 × 10^5^ cells were resuspended with 300 μl 1 × binding buffer. Subsequently, cells were stained with Annexin V-FITC and PI for 15 min in the dark and detected by flow cytometer (BD FACS Verse, USA). The results were analyzed by Flow Jo vX.0.7 software (Flow Jo, USA).

### Small interfering RNA

To investigate the role of XDH in RCC cells, XDH was knocked down by transient transfection of small interfering RNA (siRNA). Three independent *XDH* siRNA oligonucleotides and non-targeting sequences (negative controls) (Ribo Bio, China) were transfected into ACHN and 786-O cells using lipofectamine 2000 and 50 nmol/L of each siRNA according to the manufacturer's protocol. The medium containing siRNAs were replaced 4-6 h after transfection.

Total RNA and protein were extracted after transfection at 24 h and 48 h, respectively. The knockdown of gene expression was assessed by western blot and qRT‐PCR. The sequences of *XDH* siRNA were listed in Table 2.

### Xenograft tumor growth

Animal experiments complied with the ARRIVE guidelines and were carried out in accordance with the National Institutes of Health guide for the care and use of Laboratory animals (NIH Publications No. 8023, revised 1978). And animal experiments were performed in the Animal Research Facility of the Second Hospital of Tianjin Medical University. Healthy BALB/c Nude mice (6-8 weeks old, male: female = 1:1) were purchased from Beijing Vital River Laboratory Animal Technology Co., Ltd (Beijing, China). They were kept in squirrel cages, and received water and food ad libitum, during a 12 h light/dark cycle.

Mice were randomly divided into a control group (ACHN-pCDH-luc) and an experimental group (ACHN-pCDH-C1QBP-luc) (n = 10 each group), and were marked. Luciferase-labeled C1QBP overexpression and control of ACHN cells were stably constructed through lentivirus-mediated luciferase plasmids (ACHN-pCDH-luc and ACHN-pCDH-C1QBP-luc). And the efficiencies of transduction including luminescence intensity and protein expression were verified using microplate reader and western blot technology, respectively. Mice were anesthetized using pentobarbital sodium and surgery was performed to expose the left kidney on the back of the mouse. Subsequently, tumor cells (1 × 10^6^ cells, mixed with Matrigel, 1:1) were injected under the renal capsule, and incisions were sutured. The experimental units that died during the operation were excluded. Eight weeks later, mice were intraperitoneally injected with luciferin (30 µg per mouse) and detected bioluminescence signals of primary tumors by the live IVIS imaging system (Perkin Elmer, USA) (n = 6 each group). Mice were humanely killed by overdose of anesthesia, and tumors of the left kidney were removed. The weight of the primary tumor of mice was calculated by using minus the weight of the corresponding right kidney (n = 6 each group). Primary RCC tumors, were fixed in formalin, embedded in paraffin, and then cut into sections. The protein expressions in sections of primary RCC tumors were observed by IHC staining with antibodies of C1QBP, XDH, caspase-3, bax, and bcl2. The difference between the two groups was analyzed using Student's *t*-test. The blind method was used for the experiment. Non-results reader was aware of the group allocation at the stages of the outcome assessment and the data analysis. The chosen numbers per group were based on findings in previous studies and a subsequent samples size analysis.

### Statistical analysis

Statistical analysis was performed by GraphPad Prism 7.0 and SPSS 21.0 software. All data were represented as the mean ± SD. Differences between the two groups were used *t*-test. Correlation analysis between C1QBP and XDH expression was analyzed with the Spearman test. χ^2^ test was used for the analysis of the correlations between protein expression and clinicopathologic features. P < 0.05 was considered statistically significant.

## Results

### C1QBP regulates hypoxanthine level in RCC

**Figure 1:**
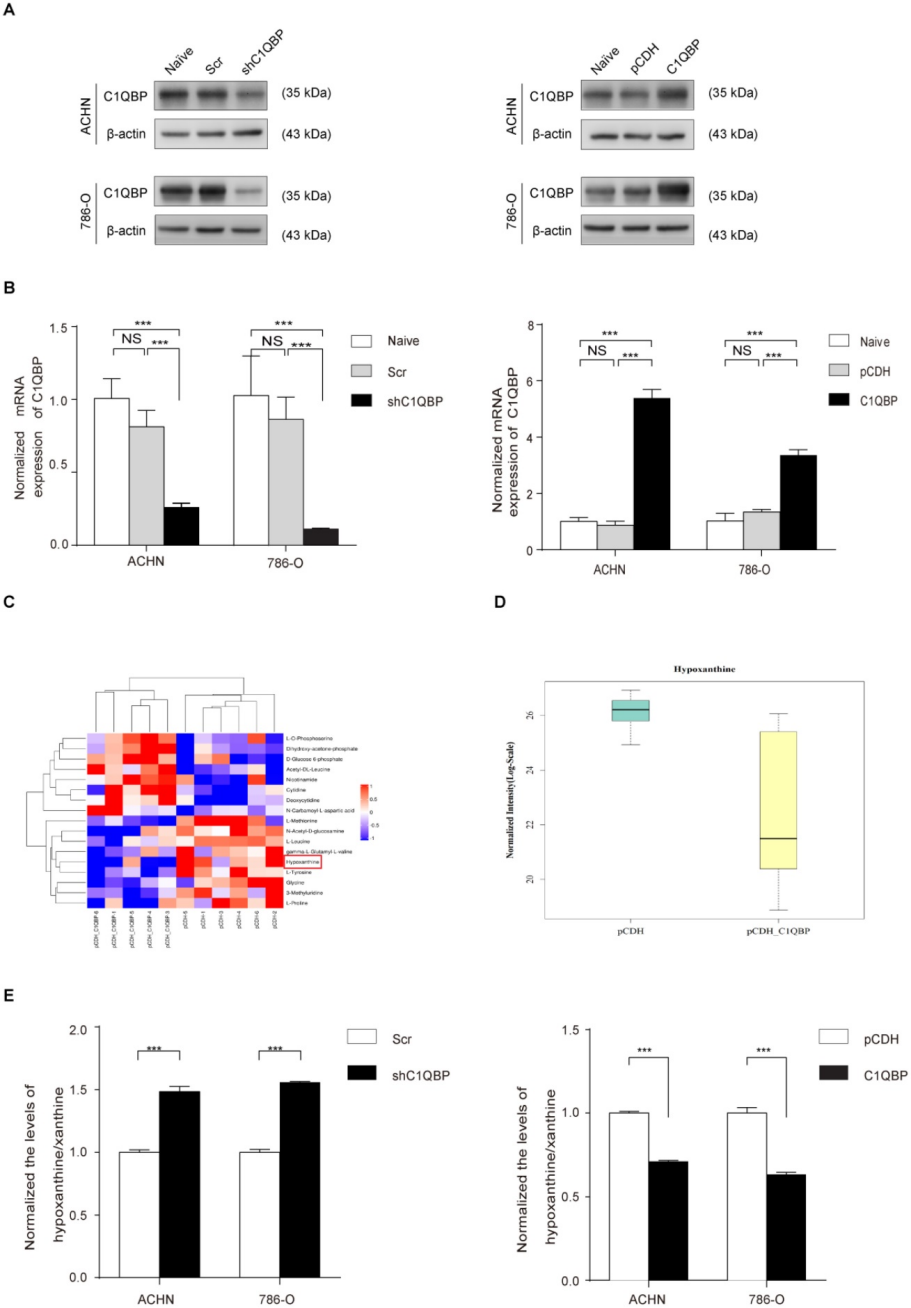
** C1QBP regulates hypoxanthine level in RCC.** ACHN and 786-O cells were transduced with lentivirus-mediated Scr control, shC1QBP or pCDH control, pCDH-C1QBP, and the expression of C1QBP was examined by western blot (A) and qRT-PCR (B). (C) According to the manufacturer's instructions 10^7^ cells in each group were collected and froze with liquid nitrogen. Metabonomic techniques were used to detect the differences of 200 major metabolites involved in cell metabolism in RCC 786-O cells control group (pCDH) and C1QBP overexpression group (pCDH-C1QBP), n = 6. (D) Among the 17 differential metabolites, the change of hypoxanthine was the most significant, and decreased by 80% in 786-O cells with overexpression of C1QBP compared with the control group,* P* = 0.01, n = 6. (E) Hypoxanthine was examined by assay kit in ACHN and 786-O cells with C1QBP knockdown (left panel) and overexpression (right panel), *P* < 0.001 by* t*-test, data were presented as mean ± SD from three independent repeats. Naïve: untreated; Scr: down-expression empty plasmid control; shC1QBP: C1QBP knockdown; pCDH: overexpression empty plasmid control; C1QBP/pCDH-C1QBP: C1QBP overexpression. Statistically significant differences were indicated: ***, *P* < 0.001. NS: no significant difference.

First, we established C1QBP overexpression and knocked-down stable RCC cell lines 786-O and ACHN and then verified the expression of C1QBP by western blot and qRT-PCR (Figure 1A, 1B). To further explore the role of C1QBP in the regulation of cellular metabolism, we examined the abundance of 200 key metabolites in C1QBP overexpression 786-O cells and the corresponding control cells (n = 6) (Supplemental Table 2). Analytes were quantified by multiple reaction monitoring (MRM) in positive or negative ion modes, and determined by electrospray ionization (ESI) MRM in positive or negative ion modes, respectively. The results showed that the overexpression of C1QBP led to significant abundance changes of 17 metabolites in 786-O cells (Figure 1C; Table 3). Intriguingly, hypoxanthine was reduced by 80% in C1QBP overexpression cells compared with the control group (Figure 1D). Next, we verified the impact of C1QBP up-regulation on the level of hypoxanthine in 786-O and ACHN cells. Consistent with the metabolomics result, C1QBP overexpression significantly decreased the hypoxanthine level (Figure 1E). The above data indicated that C1QBP involved in the regulation of RCC cell metabolism and reduced hypoxanthine level.

### C1QBP regulates the expression of XDH in RCC

**Figure 2:**
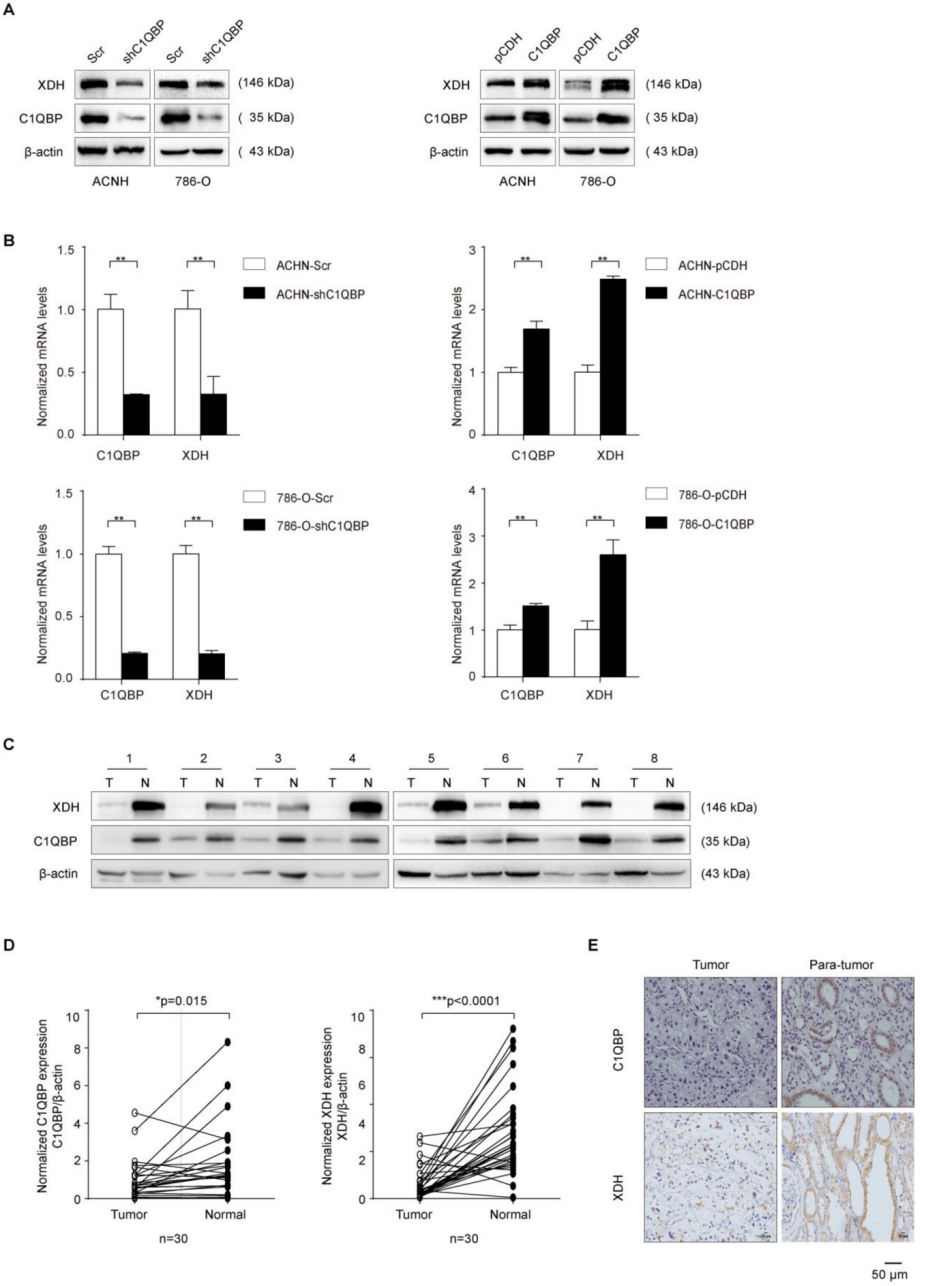
** C1QBP regulates the expression of XDH in RCC.** (A) Western blot detected the expression of XDH in ACHN and 786-O cells with stable C1QBP knockdown (left panel) and C1QBP overexpression (right panel). (B) The *XDH* mRNA was examined by qRT-PCR in ACHN (upper panel) and 786-O (lower panel) cells with stable C1QBP knockdown (left panel) and C1QBP overexpression (right panel). The expression of C1QBP and XDH was detected by western blot both in tumors (T) and adjacent normal tissues (N) of 30 primary RCC patients. (C) Representative 8 pairs of western blot images were shown. (D) The expression of C1QBP (left panel) and XDH (right panel) was normalized with β-actin and quantified by Image J software in 30 primary RCC patients. The analysis was used paired *t*-test, *P* = 0.015, *P* < 0.0001, respectively, n = 30. (E) The expression of C1QBP and XDH was examined by immunohistochemistry staining in a study cohort with 57 pairs of RCC (Tumor) and adjacent normal kidney tissues (Para-tumor). Representative pictures were shown, and brown signals indicated positive staining. Scale bar = 50μm. Statistically significant differences were indicated: *, *P* < 0.05, **, *P* < 0.01 and ***, *P* < 0.001.

XDH is a key enzyme in purine metabolism which oxidizes hypoxanthine to xanthine and catalyzes the conversion of xanthine to uric acid^12^. Previous research has shown that high tumoral XDH expression predicted poor prognosis in patients with lung adenocarcinoma^16^. We investigated whether C1QBP regulated the hypoxanthine level via XDH. The expression of XDH was detected by western blot and qRT-PCR. C1QBP knockdown decreased the mRNA and protein expression of XDH in 786-O and ACHN cells (Figure 2A, 2B). In addition, to investigate the expression of C1QBP and XDH in RCC tissues, 30 pairs of ccRCC tissues and their corresponding adjacent normal kidney tissues were examined by western blot. The results showed that C1QBP and XDH expressions were significantly (*P* = 0.015, *P* < 0.0001) decreased in the ccRCC tissues when compared with adjacent normal kidney tissues (Figure 2C, 2D). The statistical analysis showed that there was a positive correlation between the expression of C1QBP and XDH in ccRCC tissues (Table 4). Furthermore, 57 pairs of ccRCC tissues were subjected to immunohistochemical (IHC) staining and representative pictures were shown in Figure 2E. Consistent with the western blot results, IHC result showed that both C1QBP and XDH were down-regulated in ccRCC tissues, and further statistical correlation analysis revealed that C1QBP positively correlated with XDH (r = 0.552,* P* < 0.001) (Table 5).

The association of C1QBP and XDH expression with RCC clinicopathologic features was also analyzed. The result showed that the expression of C1QBP and XDH in RCC patients was both significantly (*P* < 0.05) related to patients' Fuhrman grade. In addition, C1QBP expression was related to the TNM stage of ccRCC patients. However, their expressions were not significantly associated with other clinicopathological characteristics, including gender, age, and tumor size (Table 6). Overall, the findings indicated that C1QBP positively regulated XDH expression in RCC.

### *C1QBP* modulates *XDH* mRNA at translational level

**Figure 3:**
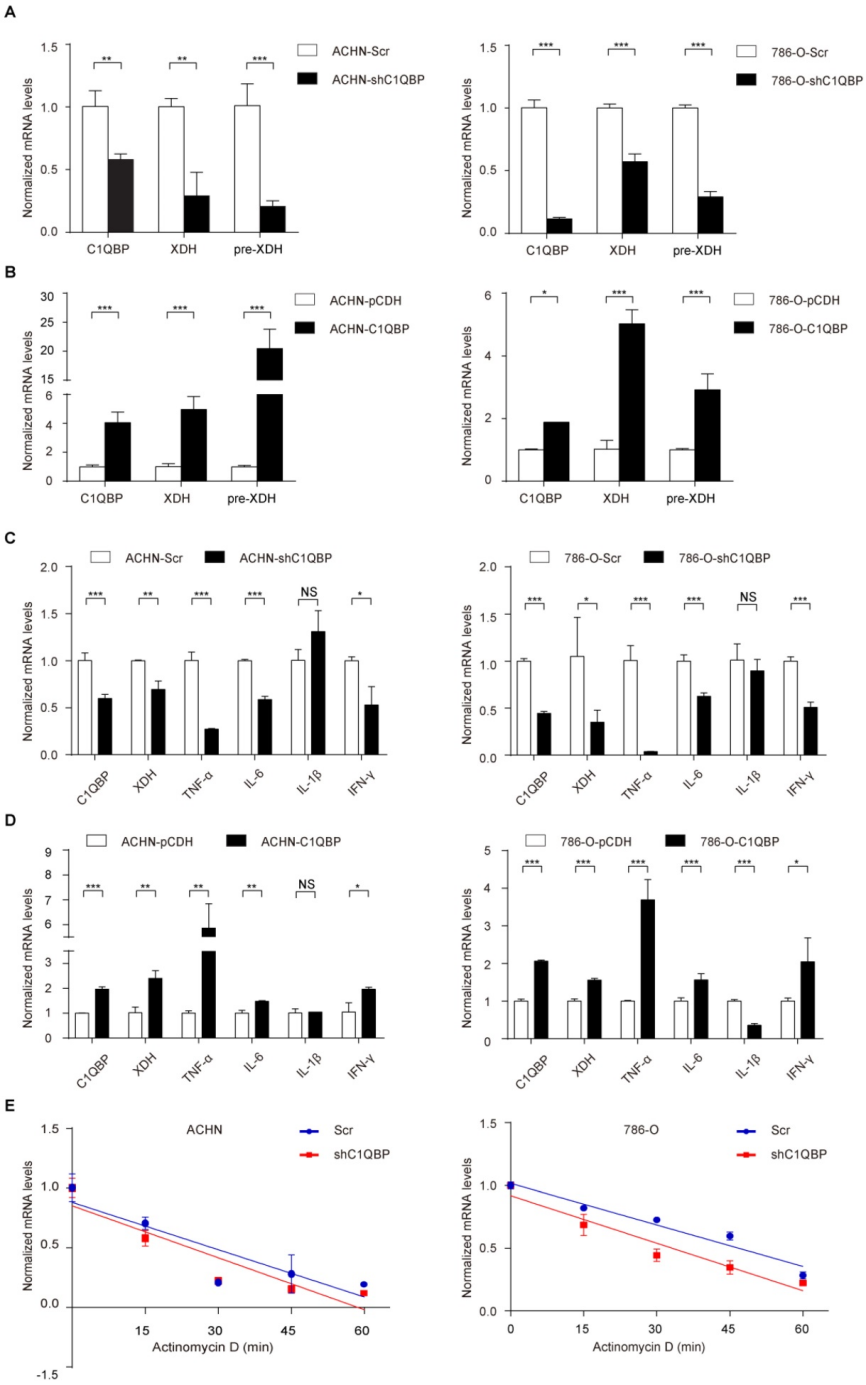
***C1QBP* modulates *XDH* mRNA at translational level.** The *XDH* pre-mRNA was examined by qRT-PCR in control group and C1QBP knockdown or overexpression group of ACHN (A) and 786-O (B) cells. *TNF-α*, *IL6*, *IL-1β*, and *IFN-γ* mRNA were examined by qRT-PCR in control group and C1QBP knockdown or overexpression group of ACHN (C) and 786-O (D) cells. (E) Control and C1QBP overexpressed ACHN and 786-O cells were treated with 5 μg/ml actinomycin D at indicated time point 0 min, 15 min, 30 min, 45 min, and 60 min. The RNA was extracted and *XDH* mRNA was detected by qRT-PCR. Statistically significant differences were indicated: *, *P* < 0.05, **, *P* < 0.01 and ***, *P* < 0.001. NS: no significant difference.

The previous study showed that C1QBP as an RNA-binding protein was involved in RNA splicing^17^. To investigate whether *C1QBP* may regulate the splicing of the *XDH* RNA, we examined the *XDH* pre-mRNA level by qRT-PCR using primers targeting junction. However, the result indicated that depletion of *C1QBP* significantly inhibited the *XDH* pre-mRNA level (Figure 3A), while the result of C1QBP overexpression was opposite (Figure 3B), suggesting that the regulation of *XDH* gene expression by *C1QBP* occurred at the transcriptional level. The expression of XDH is affected by many factors, including inflammatory cytokines, hormones, growth factors, stimuli, etc^14^. Inflammatory cytokines TNF-α**,** IL6**,** IL-1β, and IFN-γ have been reported to promote the transcription of XDH^18^. To examine whether *C1QBP* affects transcriptional stimulators of *XDH*, we measured the *TNF-α***,** *IL6***,** *IL-1β*, and *IFN-γ* mRNA. Results showed that *C1QBP* positively regulated *TNF-α*, *IL-6*, and *IFN-γ* mRNA expression in RCC cells, among which the change of *TNF-α* was the most obvious (Figure 3C, 3D). In addition, we also investigated the role of *C1QBP* on the stability of *XDH* mRNA. The results showed that the deprivation of *C1QBP* did not affect *XDH* mRNA stability in RCC cells (Figure 3E). The results indicated that *C1QBP* regulated *XDH* transcription, possibly through the regulation of the mRNA level of *XDH* transcription stimulators *IL-6*, *TNF-α*, and *IFN-γ*.

### C1QBP modulates ROS level and apoptosis in RCC

**Figure 4:**
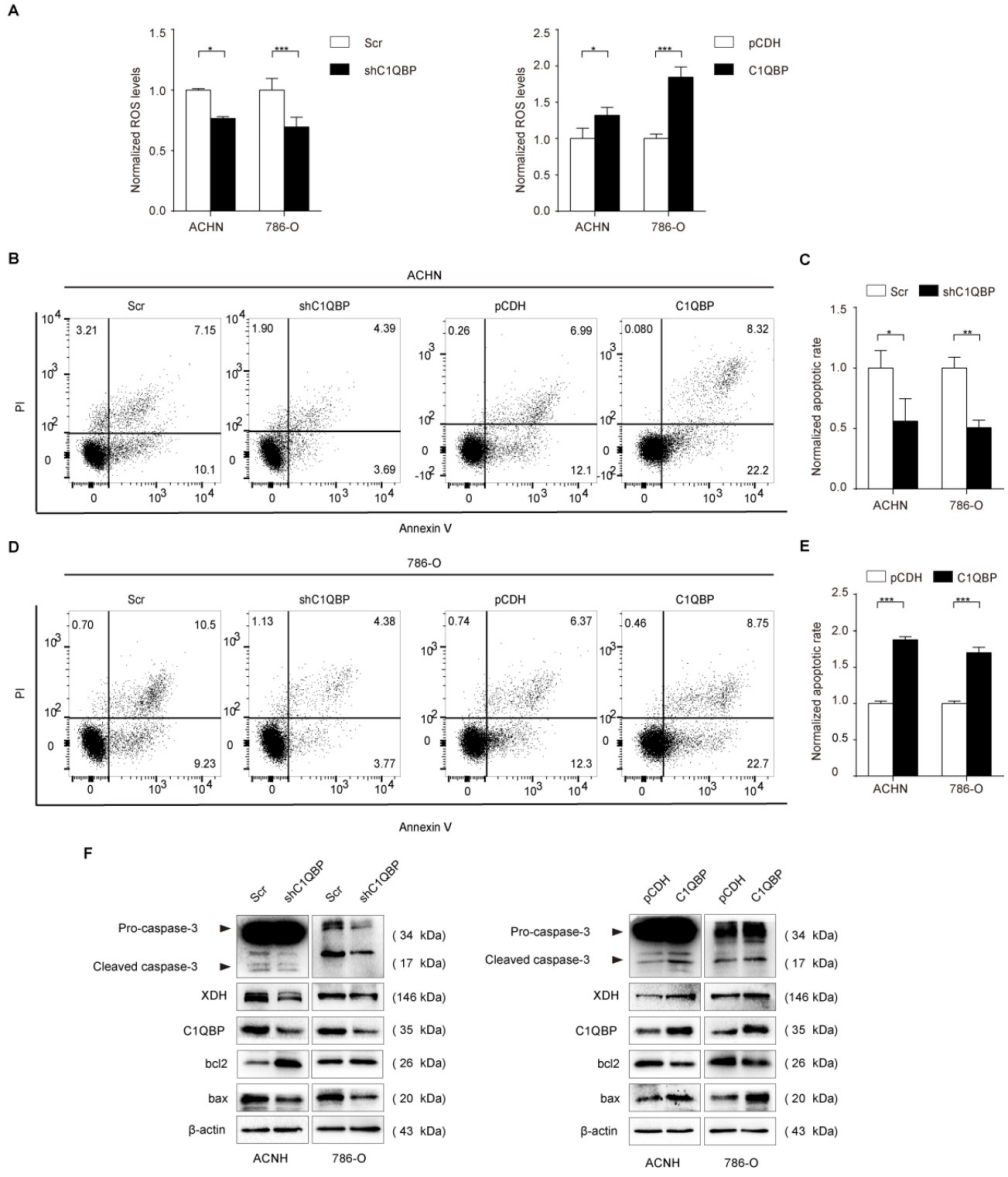
** C1QBP is critical for ROS level and apoptosis of RCC cells.** (A) In ACHN and 786-O RCC cells, ROS was examined and quantified by detecting fluorescence intensity of DCF and normalizing to control. Left panel: control and C1QBP knockdown group, right panel: control and C1QBP overexpression group. In ACHN and 786-O RCC cells, the percentage of annexin V-positive cells was determined by flow cytometry (B and D). Quantitative analysis of the apoptotic rate of cells with C1QBP knockdown (C) and C1QBP overexpression (E) was performed by normalizing to the corresponding control group. (F) The expression of C1QBP, XDH, cleaved-caspase-3, bcl2, and bax was examined by western blot in ACHN and 786-O cells with controls and C1QBP knockdown and overexpression. Data were presented as mean ± SD by *t*-test, n = 3; *, *P* < 0.05, **, *P* < 0.01, and ***, *P* < 0.001 compared to the control (Scr or pCDH).

XDH involves in the oxidative purine metabolism by catalyzing hypoxanthine to produce xanthine and ROS^19^, ^20^, and the activation of XDH generates oxidative stress^21^. We examined fluorescent probe DCF (2',7'-dichlorofluorescein, DCF) fluorescence intensity represented as ROS level in RCC cells, the results showed that DCF fluorescence was significantly decreased in C1QBP knockdown ACHN and 786-O cell lines (Figure 4A, left panel). In contrast, C1QBP overexpression increased DCF fluorescence intensity (Figure 4A, right panel). The findings indicated that C1QBP promoted ROS generation in RCC cells.

ROS played a critical role in cell apoptosis, so we investigated whether C1QBP impacted apoptosis of RCC cells (Figure 4B, 4D). We found that C1QBP downregulation inhibited cell apoptosis (Figure 4C) and C1QBP overexpression promoted cell apoptosis (Figure 4E). Meanwhile, we examined apoptosis-associated protein expression by western blot in ACHN and 786-O cells. The results demonstrated that C1QBP enhanced the expression of apoptotic protein bax and cleaved-caspase-3 and diminished anti-apoptotic protein bcl2 (Figure 4F). These results indicated that C1QBP modulated ROS generation and apoptosis of RCC cells.

### C1QBP regulates ROS and apoptosis of RCC cells via XDH

**Figure 5:**
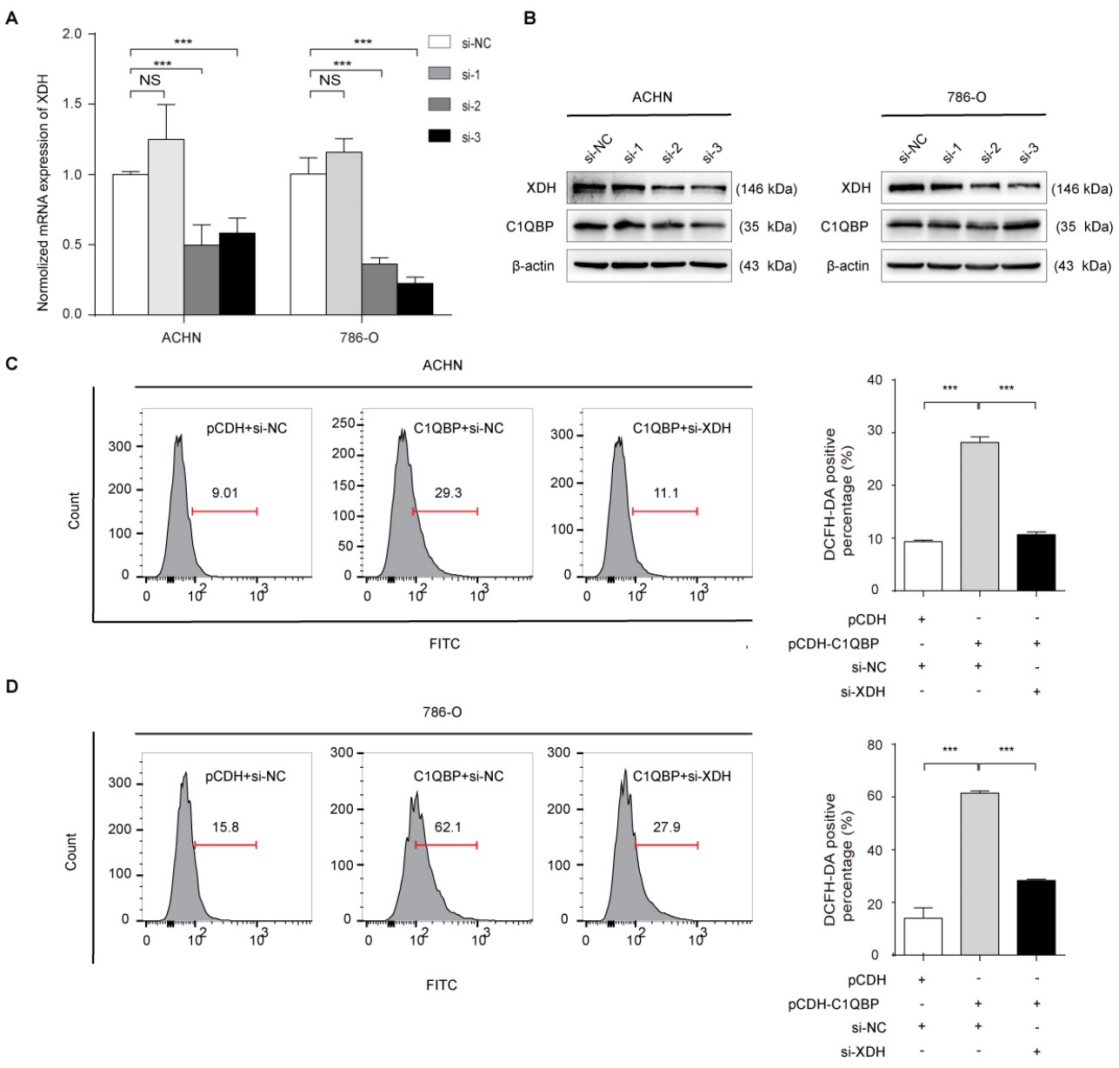

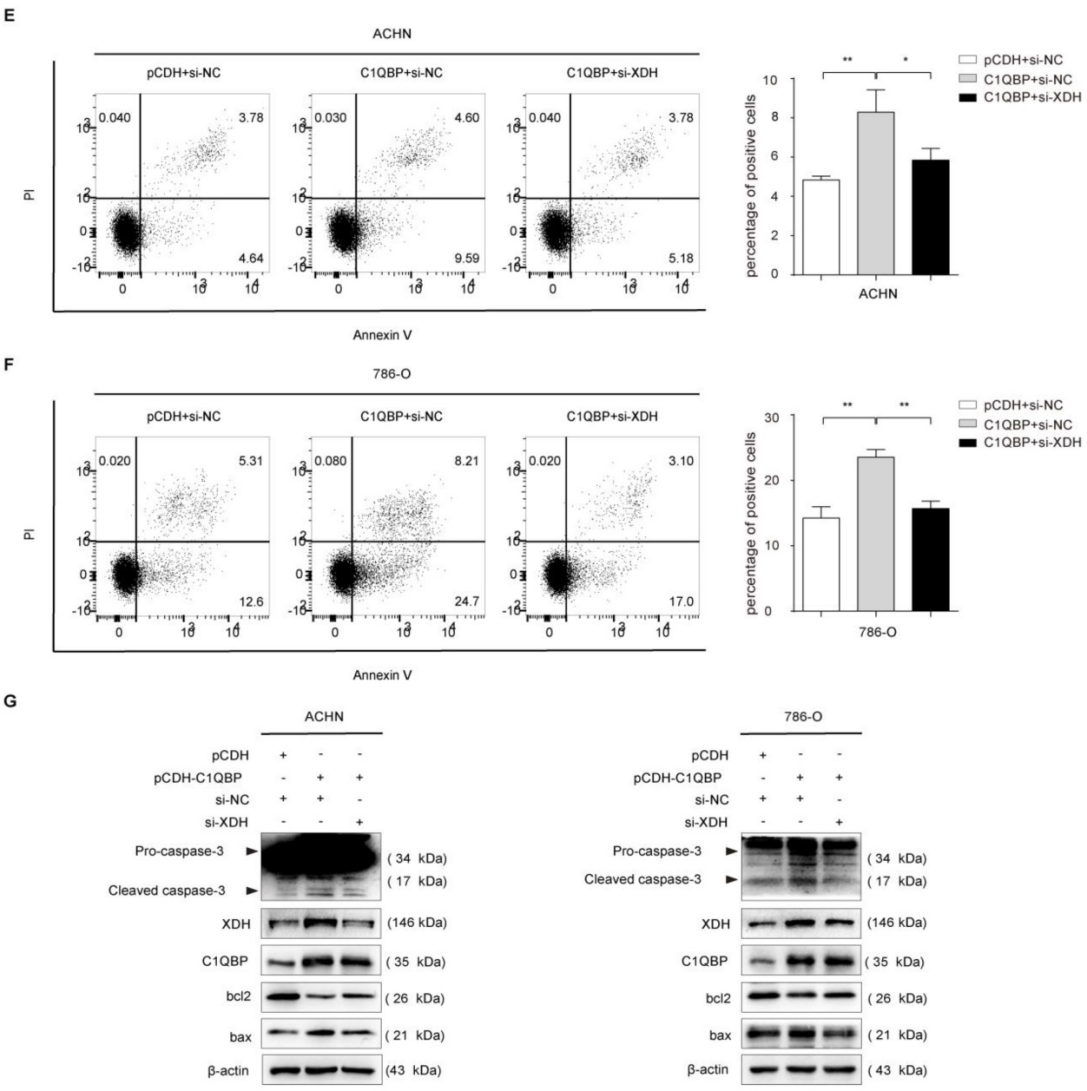
** XDH is critical for C1QBP-regulated ROS production and apoptosis of RCC.** XDH knockdown by using three independent *XDH* siRNAs (si-*XDH*-1, si-*XDH*-2, si-*XDH*-3) in ACHN and 786-O cells were evidenced by qRT-PCR (A) and western blot (B). ACHN and786-O cells were transfected with pCDH + si-NC, pCDH-C1QBP + si-NC, pCDH-C1QBP + si-*XDH*, and then ROS level (C and D) and apoptosis (E and F) were examined by flow cytometry, and (G) the expression of C1QBP, XDH, cleaved-caspase-3, bcl2, and bax was examined by western blot. β-actin was used as an internal control. Data were presented as mean ± SD by *t*-test. Statistically significant differences were indicated: *, *P* < 0.05, **, *P* < 0.01 and ***, *P* < 0.001. NS: no significant difference.

To investigate whether C1QBP regulates ROS level and cell apoptosis via modulating the expression of XDH, we did the rescue assay. As shown in Figure 5A, 5B, the third siRNA (si-*XDH*-3) was the most effective in *XDH* knockdown. In ACHN and 786-O cells with C1QBP overexpression, transfection with *XDH* siRNA partially inhibited C1QBP-induced ROS (Figure 5C, 5D) and cell apoptosis (Figure 5E, 5F). Besides, this change was consistent with the expression of the bax and cleaved-caspase-3. Corresponding deprivation of XDH partially restored the expression of the anti-apoptotic protein bcl2 (Figure 5G). These findings revealed that C1QBP regulated ROS production, cell apoptosis, and the expression of apoptosis-related proteins via regulating XDH in RCC cells.

### C1QBP overexpression suppresses RCC tumor growth and the expression of XDH and apoptosis-related proteins *in vivo*

**Figure 6:**
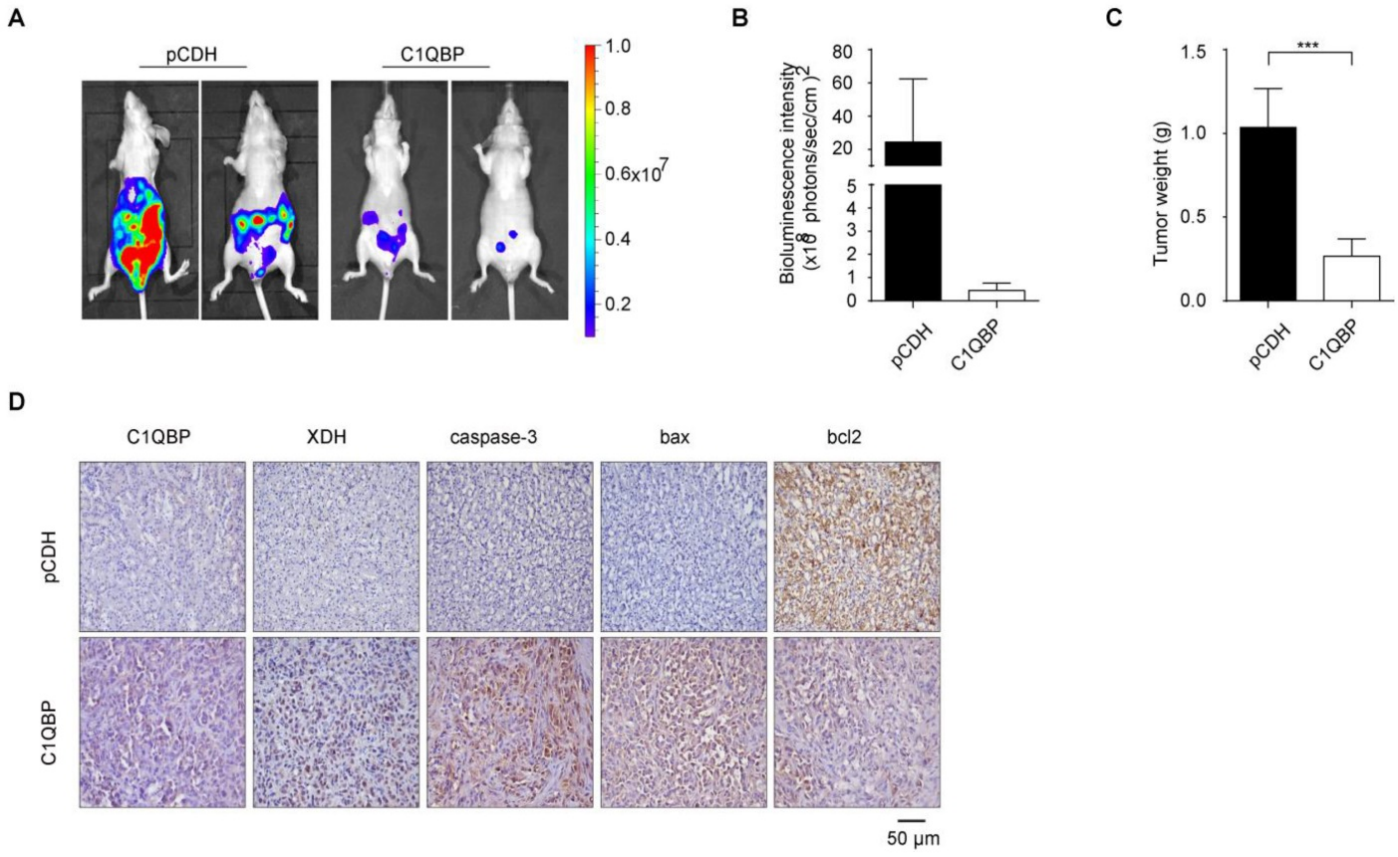
** C1QBP suppresses RCC tumor growth and regulates the expression of XDH and apoptotic proteins *in vivo***. ACHN cells transduced with luciferase-labeled C1QBP overexpression (C1QBP-luc) or control (pCDH-luc) were used to implant into BALB/c nude mice for 8 weeks, n = 6 each group. The bioluminescence was detected by the IVIS imaging system and followed by histological examination. (A) Tumor growth was examined by the IVIS imaging system, and representative images were shown. (B) Relative tumor bioluminescence intensity was quantified, n = 4 each group. (C) Left primary renal tumor weight was measured and analyzed through subtracting corresponding weight of right renal, respectively, n = 6. (D) Mice primary renal tumors were collected and processed for IHC staining of C1QBP, XDH, caspase-3, bax, and bcl2 and representative images were shown. Scale bar = 50μm. All data were presented as mean ± SD, *, *P* < 0.05, ***, *P* < 0.001 by Student's *t*-test.

To determine the role of C1QBP in the RCC progression *in vivo*, we constructed an orthotopic tumor xenograft model with implantation of stable C1QBP overexpression and control ACHN cells with luciferase into the left kidney capsule of BALB/c nude mice. After 8 weeks, mice were intraperitoneally injected with luciferin, and live imaging was performed to detect the bioluminescence intensity of the primary tumor. The results showed that mice in the C1QBP overexpression group had lower bioluminescence intensity comparing with mice in the control group (Figure 6A, 6B). Next, primary tumors were isolated from mice in these two groups, and the tumor weight was lower in the C1QBP overexpression group (Figure 6C), suggesting that C1QBP played an important role in RCC tumor growth *in vivo*. Moreover, slices of the primary renal tumor were performed for IHC staining. There was an increasing expression of XDH in the C1QBP overexpression group (Figure 6D), suggesting that C1QBP could enhance XDH expression. Additionally, in the C1QBP overexpression group, increased expression of caspase-3 and bax and decreased bcl2 expression were detected (Figure 6D). The data indicated that C1QBP regulated RCC growth *in vivo*.

## Discussion

RCC often involves systemic metastases, and the recurrence rate of RCC patients after surgery is still as high as 30%^22^. It is well-known that RCC is not sensitive to both chemotherapy and radiotherapy, and the serious adverse reactions caused by immunotherapy and the side effects and resistance of drugs in targeted therapy present challenges for the treatment of RCC^23^. Therefore, to develop new targeted anticancer drugs, in-depth research of the molecular basis of RCC progression is of great significance for improving the survival of patients with RCC. In our previous study, we found that C1QBP suppressed the progression of RCC, while the mechanism remains unclear.

Studies have shown that in liver, kidney, and breast cancer tissues, the expression of XDH is significantly lower than in normal tissues, purine catabolism is severely impaired, and anabolic enzymes are increased^24^,^25^,^26^,^27^. This change gives tumor tissue a selective growth advantage through the rescue pathway of nucleic acid biosynthesis^28^,^29^. In serous ovarian cancer, the low expression of XDH is associated with more aggressiveness and poor prognosis^30^, indicating that XDH plays an important role in cancer progression and may become a risk factor or poor prognosis.

In this study, we found that C1QBP regulated RCC cell purine catabolic metabolism via positively regulating *XDH* mRNA and protein expression in RCC cells. Furthermore, we determined that the expression of C1QBP and XDH was decreased in ccRCC comparing with corresponding adjacent normal kidney tissues, and the alteration in these proteins was associated with a higher Fuhrman grade. Mechanically, *C1QBP* stimulated the mRNA expressions of *IL-6*, *TNF*-α, and *IFN*-γ, which might promote *XDH* transcription. However, the specific in-depth mechanism has not yet been clarified. Functionally, we demonstrated that C1QBP enhanced the ROS level and apoptosis in RCC cells. We also reached consistent conclusions that C1QBP inhibited tumor growth and increased expression of XDH and pro-apoptotic related protein in mice orthotopic tumor xenografts model. Remarkably, we revealed that C1QBP promoted the production of ROS, cell apoptosis, and catabolic metabolism of hypoxanthine in RCC via regulating XDH.

Given the several important functions in multiple diseases, C1QBP has now become a promising target for the development of monoclonal antibody-based and small molecule-based therapies^5^. However, little is known about the molecular mechanism of C1QBP in the metabolism and progression of RCC. C1QBP plays critical role in mitochondria protein synthesis, maintenance of oxidative phosphorylation, and tumor metabolism reprogramming, suggesting the importance of C1QBP in metabolism^5^,^11^. In this present study, we applied metabolomics to analyze differential metabolites in C1QBP overexpression RCC cells among 200 main metabolites. Due to the low content of some metabolites, only 109 metabolites were detected of which 17 metabolites changed significantly, involving amino-acid, glucose, and nucleotide metabolism. Our results showed the content of hypoxanthine changed most significantly, so we focus on hypoxanthine. Meanwhile, we observed that the nucleotide metabolites in the upstream of hypoxanthine metabolism remained unchanged (Supplemental Table 3). Therefore, we demonstrated that C1QBP might be involved in RCC cell hypoxanthine catabolism.

XDH is strictly modulated at the transcriptional and post-translational level and highly expressed in the breast, liver, intestine, kidney, and vascular endothelial cells while lowly expressed in the lung and skin^31^. Studies have shown that the expression of XDH may be positively associated with a worse outcome in gastric and lung cancer^32^,^33^. However, XDH expression decreased in RCC is negatively associated with a high malignity grade and a worse prognosis^14^. Consistently, we got a similar conclusion that the lower XDH expression in RCC tissue was associated with higher Fuhrman grade, and there was a positive correlation between C1QBP and XDH in RCC tissues. In addition, we found that *C1QBP* positively regulated *XDH* mRNA and protein expression in RCC cells. A previous study has shown that C1QBP, as a splicing factor, can control RNA splicing by isolating necessary RNA splicing factors into inhibitory complexes^34^. While, *C1QBP* increased RCC cell *XDH* pre-mRNA level, suggesting that *C1QBP* regulated *XDH* gene expression at the transcriptional level or inhibited RNA splicing. We further demonstrated that *C1QBP* might promote *XDH* transcription via stimulating the mRNAs of *XDH* transcription stimulating factors *IL*-6, *TNF*-α, and *IFN*-γ. These results revealed that C1QBP promoted hypoxanthine catabolism via promoting the transcriptional level of *XDH*. Although pleiotropic proinflammatory cytokines TNF-α and IL-6 play important role in pathological processes such as promoting tumor aggressiveness and migration^35^,^36^,^37^. On the other hand, these cytokines are also involved in immunological processes, including inflammation and apoptosis^38^,^39^. Our research showed that these cytokines mRNAs were regulated by C1QBP in RCC cells. Inflammatory cytokines TNF-α, IL6, IL-1β, and IFN-γ have been reported to promote the transcription of *XDH*^18^. Therefore, we speculated that *C1QBP* might promote *XDH* transcription by regulating the expression of these inflammatory cytokines (TNF-α, IL6, and IFN-γ), resulting in ROS production and subsequent induction of apoptosis of RCC cells.

Oxidative stress, arising from unbalance between pro-oxidants and antioxidants, has been recognized as aging-related to lifespan^40^. ROS-driven oxidative stress has been recognized as a critical inducer of cancer cell death in response to therapeutic agents^41^. Evidence from the study has shown that ROS could promote apoptosis by activating caspase-3 in RCC cells^42^. And C1QBP causes excessive production of ROS and apoptosis in fibroblasts^43^. In this study, we revealed that C1QBP overexpression promoted ROS level and apoptosis of RCC. Oxidative stress caused by XDH promotes prostate cancer cell-specific apoptosis^21^,^44^. We further explored the effect of XDH in RCC and confirmed that the increasing ROS and apoptosis caused by C1QBP overexpression were partially restored via silencing XDH. Therefore, XDH was critical for C1QBP regulated ROS and apoptosis in RCC.

The B-cell lymphoma 2 (bcl2) family has long been identified for its important role in apoptosis^45^,^46^. Bcl2 homologous proteins contains anti-apoptotic bcl2, Mcl-1, Bcl-xL, ect. and pro-apoptotic proteins bax, bad, Beclin-1, ect^46^. Bcl-xL and Mcl-1 play a key role in survival of cancer cells, and they contribute to protection from apoptosis and promotion of cancer progress^46^,^47^. Moreover, Bcl-xL and Mcl-1 are key targets in various cancers, such as hematological cancers^47^,^48^. The apoptosis inhibitory factor bcl2 is the key regulator of apoptosis. Anti-apoptotic bcl2 and pro-apoptotic bax can form ion channels in mitochondria, resulting in changes in mitochondrial permeability (macropore formation) and the release of apoptotic protein activator cytochrome c. And the occurrence of apoptosis is accompanied by the release of cytochrome c from mitochondria, followed by the activation of caspase, which eventually leads to apoptosis^15^. The accumulation of ROS leads to an increase in the ratio of bax/bcl2 and promotes the activation of caspase-3 leading to apoptosis^45^. Our study showed that C1QBP enhanced the activation of caspase-3, the expression of bax, and inhibited the expression of bcl2 via XDH *in vivo* and *in vitro*. Our findings might imply that C1QBP affected cell apoptosis by regulating the expression of bax/bcl2 and the activation of caspase-3 which were modulated by XDH mediated ROS generation and eventually suppressed RCC progression.

In summary, the expression of C1QBP and XDH is significantly lower in RCC compared to adjacent normal tissues and associated with high Fuhrman grade. C1QBP regulates catabolism of hypoxanthine via XDH. And the regulation of *XDH* transcription by *C1QBP* may be accomplished by stimulating the mRNA of *XDH* transcription factors *IL-6*, *TNF-α*, and *IFN-γ*. In addition, C1QBP plays an important role in promoting apoptosis via regulating XDH mediated ROS generation in RCC. Our findings suggest that C1QBP is a novel mediator of RCC tumor progression and targeting C1QBP/XDH provides a potential treatment strategy for RCC. However, the exact mechanism of regulation of XDH expression by C1QBP still needs further investigation. Clinically, anti-angiogenic drugs and immunotherapy are commonly used in the treatment of RCC, but their off-target effects lead to poor therapeutic result, and the toxic stimulation of drugs can easily cause adverse reactions in patients. C1QBP and XDH as molecular biological markers will be more targeted in guiding therapy. Based on the extensive metabolic reprogramming that occurs in renal cancer, the combination of tumor molecular biology and metabolomics will be crucial for the development of new therapeutic targets for RCC, the implementation of precision medicine and the patient prognosis evaluation.

## Conclusion

We have clarified that C1QBP promotes RCC cell hypoxanthine catabolism via up-regulating XDH, and apoptosis by modulating XDH mediated ROS generation and pro-apoptotic proteins caspase-3 and bax/bcl2, then affects tumor progression. In addition, *C1QBP* promotes *XDH* pre-mRNA level, and may affect *XDH* transcription by stimulating the mRNA level of *XDH* transcriptional stimulators *IL-6*, *TNF-α*, and *IFN-γ*.

## Supplementary Material

Supplementary tables.

## Figures and Tables

**Table 1:** Primers sequences

Genes	Primers sequences (5′ to 3′)
*GAPDH*	F: GCCGTCTATGCGGCTTGT
	R: TGGAAGGGGTTCCCTGAGTT
*XDH*	F: TGCACCACCAACTGCTTAGC
	R: GGCATGGACTGTGGTCATGAG
*C1QBP*	F: AGTGCGGAAAGTTGCCGGGGA
	R: GAGCTCCACCAGCTCATCTGC
*pre-XDH*	F: CCTAGCAACCCAGCAGACTCC
	R: TCCACAGCCGAGCTTGGTTC

**Table 2:** The sequences of *XDH* siRNAs

Name	Target sequence
si-*XDH*-1	GACCTGAGCTGAAGATCGA
si-*XDH*-2	GAACTACCAGCCATTATCA
si-*XDH*-3	GCATCGTCATGAGTATGTA

**Table 3:** 17 significantly differentially expressed metabolites after C1QBP overexpression in 786-O cell

Metabolite name	Fold change	*P*-value
Hypoxanthine	**0.200**	**0.010**
N-Acetyl-D-glucosamine	0.665	0.024
L-Methionine	0.704	0.006
L-Leucine	0.723	0.041
L-Tyrosine	0.764	0.005
gamma-L-Glutamyl-L-valine	0.768	0.043
3-Methyluridine	0.867	0.018
Glycine	0.877	0.022
L-Proline	0.945	0.035
L-O-Phosphoserine	1.221	0.033
Nicotinamide	1.721	0.042
Acetyl-DL-Leucine	1.744	0.006
N-Carbamoyl-L-aspartic acid	1.926	0.047
Dihydroxy-acetone-phosphate	2.218	0.008
Cytidine	2.311	0.033
Deoxycytidine	2.450	0.012
D-Glucose 6-phosphate	2.840	0.014

**Table 4:** Clinical relevance of C1QBP and XDH in RCC by western blot

		C1QBP expression		Spearman's r Correlation coefficent	*P* value
		Up	Down		
XDH	Up	4	0	0.711	0.001**
	Down	3	23		

Note: The expression of C1QBP and XDH was quantified and compared between RCC tumor (T) and paired adjacent normal tissue (N). The relative ratio T/N > 1 was defined as “Up”, and T/N < 1 was defined as “Down”.

**Table 5:** Clinical relevance of C1QBP and XDH in RCC by IHC

		C1QBP expression		Spearman's r	*P* value
		positive	negative	Correlation coefficent	
XDH	positive	12	10	0.552	<0.001***
	negative	2	33		

Note: IHC detected the expression of protein C1QBP and XDH in 57 ccRCC tissues. Spearman's correlation coefficient analysis, ***, *P* < 0.001 difference is statistically significant.

**Table 6:** Expression of C1QBP and XDH in RCC tissues

			XDH expression		*P* value	C1QBP expression		*P* value
		N	-	+		-	+	
Gender	Male	40	26	14	0.658	29	11	0.518
	Female	17	10	7		14	3	
Age	<65	30	19	11	0.977	24	6	0.54
	≥65	27	17	10		19	8	
Tumor size	<4	12	8	4		10	2	
	≥4, ≤7	16	10	6	0.886	11	5	0.673
	>7	29	17	12		22	7	
TNM stage	T1-2	40	25	15	0.794	27	13	0.044*
	T3-4	17	10	7		16	1	
Fuhrman grade	I,II	31	15	16	0.028*	19	12	0.012*
	III,IV	26	20	6		24	2	
Location	Carcinorma	57	35	22	<0.001***	43	14	<0.001***
	Para-Carcinorma cinorma	57	4	53		13	44	

Note: IHC staining results (+): positive, (-): negative.

## Abbreviations

C1QBP: complement component 1 Q subcomponent binding protein
ccRCC: clear cell renal cell carcinoma
CO_2_: carbon dioxide
IHC: immunohistochemistry
MRM: multiple reaction monitoring
OXPHOS: oxidative phosphorylation
PCR: polymerase chain reaction
ROS: reactive oxygen species
RCC: renal cell carcinoma
shRNA: short hairpin RNA
siRNA: small interfering RNA
TNFR1: tumor necrosis factor receptor 1
TNM: tumor, node, metastasis
XDH: xanthine dehydrogenase
XOR: xanthine oxidoreductase
XO: xanthine oxidase
Bcl2: B-cell lymphoma 2
