# Supplementary material for: C1QBP regulates apoptosis of renal cell carcinoma via modulating xanthine dehydrogenase (XDH) mediated ROS generation
Source: Int J Med Sci. 2022 May 9;19(5):842–57. doi: 10.7150/ijms.71703 (PMC9149634; doi:10.7150/ijms.71703)
Supplement: Supplementary file 1 — Supplementary tables. [file ijmsv19p0842s1.pdf]

Summary

The primer sequences of pLKO.1-shC1QBP**,** MRM analysis and upstream nucleotide metabolites of hypoxanthine metabolism were provided in supplementary.

Supplemental Table 1 The primer sequences of pLKO.1-shC1QBP

	Primers sequences (5′ to 3′)
1	F:CCGG**ATGACAGTCCAACACAAGGGCCTTC**CTCGAG**GAAGGCCCTTGTGTTGGACTGTCAT**TTTTTG
	R:AATTCAAAAA**ATGACAGTCCAACACAAGGGCCTTC**CTCGAG**GAAGGCCCTTGTGTTGGACTGTCAT**
2	F:CCGG**TATCCTTCCATTCAGACTCGCCAGT**CTCGAG**ACTGGCGAGTCTGAATGGAAGGATA**TTTTTG
	R:AATTCAAAAA**TATCCTTCCATTCAGACTCGCCAGT**CTCGAG**ACTGGCGAGTCTGAATGGAAGGATA**
3	F:CCGG**TTAGGTGGTCATATAAGGCCCAGTC**CTCGAG**GACTGGGCCTTATATGACCACCTAA**TTTTTG
	R:AATTCAAAAA**TTAGGTGGTCATATAAGGCCCAGTC**CTCGAG**GACTGGGCCTTATATGACCACCTAA**

Note: The underlined part of the table is the specific interference sequence of *C1QBP* gene

Supplemental Table 2 MRM analysis of RCC cells metabolism

Metabolite name	QCRSD	p-value	Fold change
L-Tyrosine	0.02094811	0.005173707	0.763979856
L-Methionine	0.01580671	0.005675976	0.704032614
Acetyl-DL-Leucine	0.19502343	0.005882066	1.744411416
Dihydroxy-acetone-phosphate	0.03833635	0.008321489	2.217877482
Hypoxanthine	0.01556628	0.009968181	0.200368397
Deoxycytidine	0.02833936	0.011724878	2.450454125
D-Glucose 6-phosphate	0.02782175	0.013925325	2.839767942
3-Methyluridine	0.06554353	0.017837435	0.867457476
Glycine	0.05086946	0.021774133	0.87711949
N-Acetyl-D-glucosamine	0.0945042	0.023717547	0.665194119
Cytidine	0.01781936	0.032701396	2.310816596
L-O-Phosphoserine	0.05210775	0.033035939	1.22067683
L-Proline	0.01076645	0.034913344	0.94482937
L-Leucine	0.08300935	0.040979128	0.723127179
Nicotinamide	0.02110568	0.04206159	1.721276088
gamma-L-Glutamyl-L-valine	0.10339134	0.043029095	0.767516269
N-Carbamoyl-L-aspartic acid	0.20572391	0.046711403	1.925804978
Deoxyguanosine	0.22541814	0.051525631	0.393792777
Methylguanidine	0.06049732	0.055262495	1.362579355
Guanosine	0.05630341	0.055965035	0.665574212
Folic acid	0.06705206	0.062823312	0.402048951

Acetylcholine	0.06468563	0.074951227	1.718688673
Dimethylglycine	0.15924714	0.080140813	0.663932015
Flavone	0.17466823	0.08505582	0.62340973
Glycocholic acid	0.23621031	0.086870996	2.707246939
p-Hydroxybenzoic acid	0.04147678	0.090660685	0.184562991
Cytosine	0.16243533	0.090720827	1.538991924
Cytidine diphosphate choline (CDPcholine)	0.10577083	0.09337803	0.552124631
Thiamine	0.12743749	0.102355411	1.589824823
L-Homoserine	0.09086372	0.111856876	0.914881687
Imidazole	0.0567174	0.124321262	0.890224275
Deoxyinosine	0.05548109	0.125368744	0.447315085
Glycyl-L-leucine	0.11364688	0.126954806	0.614613201
S-Lactoylglutathione	0.10965808	0.127533877	3.513635509
Pantothenic acid	0.09449212	0.127538426	0.722316603
L-Kynurenine	0.05410462	0.129111684	0.714222875
N-Acetylaspartylglutamic acid (NAAG)	0.06915259	0.140126489	0.828659262
D-glucosamine 1-phosphate	0.19719578	0.146445032	1.563465167
Succinic acid	0.07039667	0.170091716	0.570794454
Nicotinic acid	0.09641147	0.178600467	0.700943809
2'-O-methyladenosine	0.06094974	0.181223789	2.240389424
Uridine 5'-monophosphate (UMP)	0.12265004	0.194008846	1.492586851
L-Phenylalanine	0.12013704	0.21792728	1.206834938
Adenosine monophosphate (AMP)	0.14976594	0.219952483	1.947801389
Allantoic acid	0.13985036	0.230330845	1.227718657
5'-Deoxyadenosine	0.10457951	0.236792969	1.473239712
gamma-L-Glutamyl-L-phenylalanine	0.01516164	0.244247996	0.80244918
Xanthosine	0.0810861	0.261226732	0.81747788
Cholesterol sulfate	0.08738028	0.277841261	1.444085894
Uridine	0.13769874	0.283535241	0.786105138
L-Tryptophan	0.09800896	0.294538965	0.843968336
Adenosine 3',5'-cyclic monophosphate (cAMP)	0.04684437	0.299412911	2.730305938
GDP-L-fucose	0.10042203	0.314168428	1.293656459
gamma-Aminobutyric acid (GABA)	0.07022735	0.320005464	1.660940754
Inosine 5'-monophosphate (IMP)	0.15860897	0.325585397	1.710431612
L-Glutamine	0.04490932	0.326161358	1.252960894
Deoxycytidine monophosphate (dCMP)	0.10545422	0.326569677	1.133981639
Nicotinamide adenine dinucleotide phosphate (NADP)	0.1005741	0.32950416	2.019066278
Alpha-N-Phenylacetyl-L-glutamine	0.17665739	0.34175727	0.038989546
sn-Glycerol 3-phosphate	0.07832303	0.341918884	0.611379851

O-Succinyl-L-homoserine	0.03857083	0.367258826	1.10229984
N-Acetyl-L-tyrosine	0.17547971	0.395056642	0.868423831
Adenosine	0.21146821	0.400028931	0.830746892
Adenosine 5'-phosphosulfate (APS)	0.10644329	0.400706891	3.398676719
L-Glutamic acid	0.07048518	0.412768379	0.317147407
Pseudouridine	0.0092023	0.420987735	0.686751661
myo-Inositol	0.00999448	0.424855333	0.817662625
N-Acetylglutamine	0.14826431	0.427442206	0.68116836
L-2-Hydroxygluterate	0.0522647	0.440206976	1.142454924
Sarcosine	0.01199709	0.440617763	1.081297268
7-methylguanosine	0.26623719	0.461914346	1.393412352
N-Acetyl-L-alanine	0.16430756	0.463234684	1.078161875
N6-methyladenosine	0.0286924	0.46996747	0.831429389
4-Pyridoxic acid	0.06837403	0.482348505	1.237183175
Thymine	0.09108947	0.48292546	0.358170095
Fumaric acid	0.07627195	0.494568916	1.13440706
Sepiapterin	0.23230619	0.502580696	0.841917661
Nicotinamide adenine dinucleotide (NAD)	0.01790487	0.508489006	0.941435437
Deoxycytidine triphosphate (dCTP)	0.06466501	0.511347627	1.0993817
Cytidine 5'-monophosphate (CMP)	0.05888228	0.515254009	0.84581602
Cytidine triphosphate (CTP)	0.15190542	0.552806706	1.556728454
N2,N2-Dimethylguanosine	0.02902064	0.575947274	0.687509022
Uridine 5'-diphospho-glucuronic acid (UDP-D-Glucuronate)	0.13109979	0.595508499	0.908850974
N-Acetylcadaverine	0.19294534	0.62014993	1.06509275
Riboflavin (Vitamin B2)	0.06114656	0.627138453	0.832333215
N-Acetylputrescine	0.04943184	0.634824774	1.05677407
Pyridoxal (Vitamin B6)	0.07454038	0.651708367	0.843610309
Nicotinic acid adenine dinucleotide (NAAD)	0.05342037	0.661692378	0.942881275
D-Fructose 1,6-bisphosphate	0.20179483	0.674906095	0.74992467
L-Carnosine	0.01279468	0.70269574	1.071687109
Inosine	0.02479195	0.70801646	1.079157244
Malic acid	0.19630838	0.709107778	0.936120204
Creatinine	0.03438992	0.712980149	1.036965953
Orotic acid	0.1604694	0.727456396	1.125340152
Glycerophosphocholine	0.06031577	0.742425765	1.192611602
Flavin adenine dinucleotide (FAD)	0.11638122	0.788606194	1.174551939
Argininosuccinic acid	0.22203372	0.814475546	0.956076171
Pyridoxine	0.20524857	0.824094247	1.084723219
cis-4-Hydroxy-D-proline	0.11058885	0.848530625	1.015302196
Creatine	0.00669503	0.889417071	0.99846091
Glyceric acid	0.26381111	0.892718252	1.064974679

N4-Acetylcytidine	0.06174598	0.904662245	0.984021023
Urocanic acid	0.07911292	0.909297062	0.972520148
L-Carnitine	0.05968643	0.922242277	0.995009903
N6-Acetyl-L-lysine	0.26845362	0.937576498	1.010285041
L-Dihydroorotic acid	0.09076196	0.944760745	1.022952205
Uridine diphosphate glucose (UDP-D-Glucose)	0.03076776	0.96853304	1.002411468
4-Guanidinobutyric acid	0.03839846	0.97326788	0.995874034
Deoxyadenosine monophosphate (dAMP)	0.16222032	0.993630282	1.001730933

Supplemental Table 3 Upstream nucleotide metabolites related to hypoxanthine metabolism

Metabolite name	Fold change	*P* value
Adenosine monophosphate (AMP)	0.220	1.948
Adenosine	0.400	0.831
Inosine 5'-monophosphate (IMP)	0.326	1.710
Inosine	0.708	1.079
Xanthosine	0.261	0.817
